# Supplementary material for: Within-Person Modulation of Neural Networks following Interoceptive Awareness Training through Mindful Awareness in Body-Oriented Therapy (MABT): A Pilot Study
Source: Brain Sci. 2023 Sep 30;13(10):1396. doi: 10.3390/brainsci13101396 (PMC10605589; doi:10.3390/brainsci13101396)
Supplement: Supplementary file 1 [file brainsci-13-01396-s001.zip › Supplementary Table S1.pdf]

Table S1. Intervention Effects on Self-Report Measures

	Group			Time			Group * Time		
**Measure**	**β**	**95% CI**	***p***	**β**	**95% CI**	***p***	**β**	**95% CI**	***p***
Interoception (MAIA)	-.05	[-.77, .67]	.884	.36	[-.15, .86]	.162	**1.10**	**[.39, 1.82]**	**.003**
Attention Regulation	-.15	[-.89, .60]	.693	.23	[-.27, .73]	.354	**1.18**	**[.47, 1.88]**	**.002**
Body Listening	-.03	[-.75, .70]	.944	.41	[-.19, 1.00]	.177	**1.01**	**[.17, 1.86]**	**.020**
Emotional Awareness	-.25	[-1.09, .60]	.558	.21	[-.30, .71]	.408	.70	[-.01, 1.41]	.054
Not Distracting	-.34	[-1.18, .49]	.409	-.23	[-.70, .24]	.333	**1.15**	**[.48, 1.82]**	**.001**
Noticing	.00	[-.78, .78]	1.000	.36	[-.21, .93]	.205	**.83**	**[.03, 1.63]**	**.043**
Not Worrying	.58	[-.23, 1.39]	.154	.29	[-.31, .89]	.336	.29	[-.56, 1.14]	.495
Self Regulation	.10	[-.59, .79]	.765	.56	[-.02, 1.14]	.060	**.93**	**[.11, 1.75]**	**.028**
Trusting	.02	[-.83, .88]	.955	-.02	[-.55, .50]	.926	.68	[-.06, 1.42]	.072
Symptom Burden									
Anxiety (GAD-7)	-.32	[-1.19, .54]	.456	**-.68**	**[-1.25, -.11]**	**.021**	.45	[-.35, 1.26]	.263
Depression (PHQ-9)	.35	[-.53, 1.23]	.428	.06	[-.42, .53]	.815	-.51	[-1.19, .16]	.129
Somatic (PHQ-15)	-.04	[-.93, .85]	.925	.02	[-.51, .55]	.937	.21	[-.54, .96]	.575
Stress (PSS)	-.27	[-1.09, .55]	.506	**-.85**	**[-1.29, -.40]**	**.001**	.18	[-.45, .81]	.564
Mindfulness Skills									
Decentering (EQ)	.68	[-.10, 1.46]	.084	**.57**	**[.04, 1.11]**	**.037**	.13	[-.63, .89]	.724
Non-Judgment (FFMQ)	.53	[-.28, 1.33]	.193	.55	[-.03, 1.13]	.063	.14	[-.68, .97]	.726

Note. This table presents the results of the multilevel models exploring the effects of Group [Control vs. MABT] and Time [Baseline vs. Post-Intervention] on the self-report measures. 95% CI = 95% confidence interval around the estimated parameter. Parameters with $p < .05$ are highlighted in bold.
