# Supplementary material for: Within-Person Modulation of Neural Networks following Interoceptive Awareness Training through Mindful Awareness in Body-Oriented Therapy (MABT): A Pilot Study
Source: Brain Sci. 2023 Sep 30;13(10):1396. doi: 10.3390/brainsci13101396 (PMC10605589; doi:10.3390/brainsci13101396)
Supplement: Supplementary file 1 [file brainsci-13-01396-s001.zip › Supplementary Table S2.pdf]

Table S2. Self-Report Measures, Group, and Connectivity Correlations.

Variable	*M*	*SD*	1	2	3	4	5	6	7	8	9
1. Group [MABT]	1.50	0.51									
2. DAN / Insula	0.00	0.04	.76**<br>[.50, .90]								
3. MAIA / Somatosensory	0.01	0.03	.27<br>[-.17, .62]	.04<br>[-.39, .45]							
4. Anxiety (GAD7)	-2.18	4.53	.25<br>[-.20, .61]	.09<br>[-.34, .49]	.30<br>[-.14, .64]						
5. Depression (PHQ9)	-1.00	3.98	-.33<br>[-.66, .11]	-.26<br>[-.62, .18]	-.14<br>[-.53, .30]	.51*<br>[.12, .77]					
6. Somatic (PHQ15)	0.55	3.71	.13<br>[-.31, .52]	.22<br>[-.22, .59]	-.35<br>[-.67, .08]	.49*<br>[.08, .75]	.48*<br>[.07, .75]				
7. Decentering (EQ)	4.36	5.88	.08<br>[-.35, .48]	.14<br>[-.30, .53]	.20<br>[-.24, .57]	-.01<br>[-.43, .42]	-.32<br>[-.65, .12]	-.31<br>[-.64, .13]			
8. Stress (PSS)	-2.27	2.16	.13<br>[-.31, .52]	-.05<br>[-.46, .38]	.11<br>[-.33, .51]	.56**<br>[.18, .79]	.45*<br>[.03, .73]	.59**<br>[.23, .81]	-.55**<br>[-.79, -.17]		
9. Nonjudgement	4.73	7.09	.08<br>[-.35, .48]	.11<br>[-.33, .51]	.22<br>[-.23, .58]	-.23<br>[-.59, .21]	-.43*<br>[-.72, -.01]	-.45*<br>[-.73, -.04]	.65**<br>[.31, .84]	-.54**<br>[-.78, -.15]	
10. MAIA	5.72	6.58	.58**<br>[.21, .80]	.58**<br>[.21, .81]	.55**<br>[.16, .79]	.31<br>[-.13, .65]	-.25<br>[-.61, .19]	-.10<br>[-.50, .34]	.59**<br>[.22, .81]	-.12<br>[-.52, .31]	.37<br>[-.06, .69]

Note. The relationship between the Self-Report Measures with Group, and the two regions of interest. Means, standard deviations, and correlations with confidence intervals. *M* and *SD* are used to represent mean and standard deviation, respectively. Values in square brackets indicate the 95% confidence interval for each correlation. The confidence interval is a plausible range of population correlations that could have caused the sample correlation (Cumming, 2014). * indicates $p < .05$. ** indicates $p < .01$.
