# Supplementary material for: Within-Person Modulation of Neural Networks following Interoceptive Awareness Training through Mindful Awareness in Body-Oriented Therapy (MABT): A Pilot Study
Source: Brain Sci. 2023 Sep 30;13(10):1396. doi: 10.3390/brainsci13101396 (PMC10605589; doi:10.3390/brainsci13101396)
Supplement: Supplementary file 1 [file brainsci-13-01396-s001.zip › Supplementary Table S3.pdf]

Table S3. MAIA, Group, and Connectivity Correlations.

Variable	*M*	*SD*	1	2	3	4	5	6	7	8	9	10	11
1. Group [MABT]	1.50	0.51											
2. DAN / Insula	0.00	0.04	.76**<br>[.50, .90]										
3. MAIA / Somatosensory	0.01	0.03	.27<br>[-.17, .62]	.04<br>[-.39, .45]									
4. MAIA	5.72	6.58	.58**<br>[.21, .80]	.58**<br>[.21, .81]	.55**<br>[.16, .79]								
5. Noticing	0.83	1.07	.42*<br>[.00, .72]	.27<br>[-.18, .62]	.48*<br>[.08, .75]	.76**<br>[.50, .89]							
6. Not Distracting	0.36	1.01	.61**<br>[.26, .82]	.40<br>[-.02, .70]	.23<br>[-.21, .60]	.57**<br>[.20, .80]	.28<br>[-.16, .63]						
7. Not Worrying	0.50	1.12	.15<br>[-.29, .54]	.29<br>[-.15, .64]	.27<br>[-.17, .62]	.74**<br>[.46, .89]	.54**<br>[.15, .78]	.33<br>[-.11, .66]					
8. Attention Regulation	0.88	1.07	.60**<br>[.24, .82]	.53*<br>[.14, .78]	.53*<br>[.14, .78]	.89**<br>[.75, .95]	.65**<br>[.31, .84]	.58**<br>[.20, .80]	.63**<br>[.29, .83]				
9. Emotional Awareness	0.54	0.85	.41<br>[-.02, .71]	.51*<br>[.12, .77]	.13<br>[-.31, .53]	.60**<br>[.24, .81]	.48*<br>[.07, .75]	.13<br>[-.31, .52]	.33<br>[-.11, .66]	.44*<br>[.02, .73]			
10. Self Regulation	1.12	1.15	.46*<br>[.04, .74]	.54*<br>[.15, .78]	.58**<br>[.21, .81]	.85**<br>[.68, .94]	.56**<br>[.19, .80]	.32<br>[-.11, .66]	.64**<br>[.29, .83]	.81**<br>[.59, .92]	.54**<br>[.15, .78]		
11. Body Listening	1.09	1.30	.48*<br>[.07, .75]	.50*<br>[.09, .76]	.58**<br>[.20, .80]	.89**<br>[.75, .95]	.57**<br>[.20, .80]	.51*<br>[.11, .77]	.53*<br>[.14, .78]	.75**<br>[.48, .89]	.52*<br>[.13, .77]	.78**<br>[.54, .91]	
12. Trusting	0.39	1.13	.38<br>[-.05, .69]	.49*<br>[.08, .75]	.40<br>[-.02, .71]	.67**<br>[.35, .85]	.49*<br>[.09, .76]	.29<br>[-.15, .63]	.42<br>[-.01, .71]	.46*<br>[.04, .74]	.28<br>[-.16, .63]	.43*<br>[.01, .72]	.60**<br>[.24, .82]

Note. The relationship between the MAIA and its subscales with Group, and the two regions of interest. Means, standard deviations, and correlations with confidence intervals. For the Group variable, Control = 0, MABT = 1. *M* and *SD* are used to represent mean and standard deviation, respectively. Values in square brackets indicate the 95% confidence interval for each correlation. The confidence interval is a plausible range of population correlations that could have caused the sample correlation (Cumming, 2014). * indicates $p < .05$. ** indicates $p < .01$.
